# Dangers of Intra-Rectal Examination

**Published:** 1875-04

**Authors:** 


Dangers of Intra-Rectal Examination.—The autopsy revealed the existence of a narrow band-like stricture at the middle of the transverse colon, admitting only the tip of the little finger, and formed by a growth in the narrow walls of the intestine, and of a character yet undetermined. Above the stricture, in the fluid contained in the colon, was found an orange pit, coated lightly with hardened fæces. As this was the only solid body found, it was inferred that it might have occasioned the final obstruction. The gall-bladder was distended by reason of a large calculus plugging up the outlet. No adhesion existed here to indicate the escape by ulceration of a similar calculus, and consequent formation of the stricture. The liver and kidneys were in a state of fatty degeneration, but the heart was normal.

On raising up the small intestines from the pelvis, about two teaspoonfuls of blood was found in Douglas' *cul-de-sac*, and running from the level of the sacro-iliac sycondrosis to the bottom of the *cul-de-sac* was seen, on the anterior aspect of the rectum, a rent involving the muscular and peritoneal coats. This longitudinal rent was really divided into two by an interposing band of longitudinal muscular fibres, but the ruptures had evidently been produced at one time.—*Medical Record.*

